# Untargeted Headspace-Gas Chromatography-Ion Mobility Spectrometry in Combination with Chemometrics for Detecting the Age of Chinese Liquor (Baijiu)

**DOI:** 10.3390/foods10112888

**Published:** 2021-11-22

**Authors:** Shuang Chen, Jialing Lu, Michael Qian, Hongkui He, Anjun Li, Jun Zhang, Xiaomei Shen, Jiangjing Gao, Yan Xu

**Affiliations:** 1 Laboratory of Brewing Microbiology and Applied Enzymology, Key Laboratory of Industrial Biotechnology of Ministry of Education, State Key Laboratory of Food Science & Technology, School of Biotechnology, Jiangnan University, Wuxi 214122, China; shuangchen@jiangnan.edu.cn (S.C.); lujialingptj@163.com (J.L.); zjchem163@163.com (J.Z.); 7160201002@vip.jiangnan.edu.cn (J.G.); 2 Department of Food Science & Technology, Oregon State University, Corvallis, OR 97331, USA; michael.qian@oregonstate.edu; 3 The Center for Solid-State Fermentation Engineering of Anhui Province, Bozhou 236820, China; hehongkui@gujing.com.cn (H.H.); lianjun@gujing.com.cn (A.L.); shenxiaomei@gujing.com.cn (X.S.)

**Keywords:** Chinese liquor (Baijiu), ageing discrimination, HS-GC-IMS, extraction condition optimization

## Abstract

This paper proposes the combination of headspace-gas chromatography-ion mobility spectrometry (HS-GC-IMS) and chemometrics as a method to detect the age of Chinese liquor (Baijiu). Headspace conditions were optimized through single-factor optimization experiments. The optimal sample preparation involved diluting Baijiu with saturated brine to 15% alcohol by volume. The sample was equilibrated at 70 °C for 30 min, and then analyzed with 200 μL of headspace gas. A total of 39 Baijiu samples from different vintages (1998–2019) were collected directly from pottery jars and analyzed using HS-GC-IMS. Partial least squares regression (PLSR) analysis was used to establish two discriminant models based on the 212 signal peaks and the 93 identified compounds. Although both models were valid, the model based on the 93 identified compounds discriminated the ages of the samples more accurately according to the goodness of fit value (R^2^) and the root mean square error of prediction (RMSEP), which were 0.9986 and 0.244, respectively. Nineteen compounds with variable importance for prediction (VIP) scores > 1, including 11 esters, 4 alcohols, and 4 aldehydes, played vital roles in the model established by the 93 identified compounds. Overall, we determined that HS-GC-IMS combined with PLSR could serve as a rapid and accurate method for detecting the age of Baijiu.

## 1. Introduction

Ageing is an integral part of the production of most distilled spirits, such as whiskey and brandy, and improves their quality [1,2]. In general, freshly distilled spirits smell and taste rough, unpleasant, and unbalanced [2,3]. During the ageing period, some compounds in the spirits undergo chemical reactions, which affect the final flavor and taste profiles of the spirits [1,2,4]. Ageing plays a critical role in the production process of high-quality spirits, but is extremely time-consuming and often requires several years or more to complete [5]. Consequently, spirits’ economic value is highly associated with their age [6]. Owing to the commercialization and relatively high costs of aged spirits, counterfeiting these products is common worldwide [7]. Therefore, it is necessary to establish a rapid and accurate method to detect the age of spirits to protect consumers from being deceived concerning the age and quality of the spirits.

Baijiu (Chinese liquor), similar to whiskey and brandy, needs to be aged to develop its high-quality flavor [8]. Fresh Baijiu, which is distilled from fermented grains, is aged by being stored in pottery jars for years [9]. During this time, various chemical reactions, including reduction–oxidation, esterification, the Maillard reaction, hydrolysis, condensation, and decomposition, proceed gradually [10]. As a result, aged Baijiu smells and tastes more delightful and harmonious than its fresh alternative [11,12]. Aroma is one of the most important characteristics of Baijiu, and is a standard indicator of the age of the beverage [13,14]. Because the ageing time of Baijiu is closely related to its quality and market price, it is necessary to detect the age of Baijiu by aromatic components to protect consumers from being deceived with regard to its age and quality. To date, a few studies have been conducted on the ageing of Baijiu. Xu et al. used a gas chromatography–flame ionization detector (GC-FID) combined with principal component analysis (PCA) and cluster analysis to characterize the changes in 21 major aroma components in Baijiu during ageing and ultimately detect its age [13]. Peng et al. developed a rapid approach to discriminate Baijiu age using a gas chromatography–flash electronic nose technique combined with PCA and discriminant factor analysis [15]. Ma et al. studied the transformation of aroma components in nine Baijiu samples, with ages ranging from 0 to 30 years, using GC-MS and GC-FID [16].

Headspace-gas chromatography-ion mobility spectrometry (HS-GC-IMS) is an emerging approach in food control that uses new gas-phase separation and detection technology [17,18,19,20]. HS-GC-IMS implements a two-dimensional separation process of volatile compounds, using a combination of GC and IMS, which facilitates the identification and differentiation of different samples [20]. In recent years, HS-GC-IMS applications have increased dramatically, including for food classification and adulteration [17,21,22,23,24,25,26], production monitoring [27], and storage monitoring [28]. Li et al. compared HS-SPME-GC-MS with HS-GC-IMS to identify the age of brandy [5]. The results indicated that the model using HS-GC-IMS and partial least squares regression (PLSR) is more effective than the GC-MS model. However, the flavor of Baijiu is relatively complex, and few studies have adopted HS-GC-IMS for evaluating Baijiu. Although HS-GC-IMS is a useful tool for detecting the age of Baijiu, it is sensitive to experimental conditions, such as NaCl additions, incubation temperature, alcohol content, and injection volume [17,18,29]. Therefore, the headspace conditions should be optimized before sample analysis to obtain the most accurate information.

Thus, this study aimed to (1) optimize the experimental conditions of HS-GC-IMS for analyzing Baijiu and (2) establish and validate a model for Baijiu age identification using the HS-GC-IMS database. Additionally, this study investigated the changes in the volatile organic compounds within the samples.

## 2. Materials and Methods

### 2.1. Baijiu Samples

Thirty-nine strong-aroma samples of Baijiu from eight different vintages were collected and stored directly in pottery jars without blending. The vintages used were 1998 (*n* = 2), 2004 (*n* = 5), 2008 (*n* = 4), 2012 (*n* = 6), 2014 (*n* = 5), 2016 (*n* = 6), 2018 (*n* = 5), and 2019 (*n* = 6). All samples were stored at 4 °C before the analysis. The samples were obtained from Anhui Gujing Distillery Co., Ltd. (Anhui, China). Additionally, the A2–1 sample was used as the reference matrix for the optimization experiments. Detailed information regarding the samples is provided in Supplementary Table S1.

### 2.2. Reagents and Standards

All analytical standards used for the identification of the aroma compounds were GC grade, with at least 95% purity. The following standards were obtained from Sigma Aldrich in Shanghai, China: (E,Z)-2,6-nonadienal; 1,1-diethoxyethane; 1-octanol; 2-pentanone; 3-methyl-1-butanol; 3-methylbutanal; 3-methylbutyl butanoate; 3-methylbutyl hexanoate; acetic acid; benzaldehyde; 1-butanol; butan-2-ol; butan-2-one; butyl acetate; ethyl 3-methylbutanoate; ethyl 4-methylpentanoate; ethyl acetate; ethyl isobutyrate; ethyl lactate; ethyl pentanoate; heptan-1-ol; hexanal; propyl hexanoate; isoamyl acetate; linalool; methyl hexanoate; octanal; octanoic acid ethyl ester; pentan-2-ol; pentyl acetate; propionaldehyde; terpinen-4-ol; and butyraldehyde. The following standards were obtained from J&K in Shanghai, China: 2-methyl butanoic acid ethyl ester, 2-methyl-1-propanol, ethyl butyrate, ethyl heptanoate, isobutyl acetate, pentyl butanoate, and propan-2-one. The following standards were obtained from Aladdin in Shanghai, China: (E)-2-hexenal, 1-hexanol, 1-methylethyl acetate, 2-heptanone, 2-methylbutanal, ethyl hexanoate, furfural, methyl 2-methylbutanoate, methylpropanal, nonanal, and propanoic acid ethyl ester. N-ketones (C_4_–C_9_) used for the calculation of retention indices (RIs) were obtained from Gesellschaft für Analytische Sensorsysteme (G.A.S., mbH, Beijing, China). Ethanol (HPLC-grade, 99.9%) was purchased from J&K Scientific. Water was purified using a Milli-Q water purification system (Millipore, Bedford, MA, USA). Sodium chloride (NaCl) was purchased from Sinopharm Chemical Reagent Co., Ltd. (Shanghai, China).

### 2.3. HS-GC-IMS Method

#### 2.3.1. Optimization of Headspace Conditions

The headspace conditions were optimized using a single-factor optimization experiment. The independent variables and levels selected for the optimization experiment were diluent (ultrapure water and saturated brine), alcohol content by volume (1%, 2%, 5%, 10%, 15, and 30%), incubation temperature (40, 50, 60, and 70 °C), and injection volume (40, 100, 200, and 300 μL).

#### 2.3.2. GC-IMS Conditions

For the HS-GC-IMS analysis, a Shimadzu GC-2010 gas chromatograph (Shimadzu, Kyoto, Japan) equipped with a Perkin Elmer TurboMatrix16 autosampler (Perkin Elmer, MA, USA.) was coupled to an IMS module from GAS (Dortmund, Germany).

The analytes were separated in a DB-FFAP column (60 m × 0.25 mm × 0.25 μm film thickness; J & W Scientific; Folsom, CA, USA.) using nitrogen gas (>99.999%) at a constant flow rate of 0.8 mL/min. The temperature of the column was maintained at 40 °C for 3 min, then was increased to 150 °C at 4 °C/min, and held at 150 °C for 5 min (total of 35.5 min).

After the analytes were separated in the column, they were driven into the IMS module. First, the volatile organic compounds were ionized by the tritium source in positive mode. Then, the ions were placed into a 9.8 cm long drift tube operating at 500 V/cm and 45 °C. Next, nitrogen drift gas (>99.999%) was introduced at 150 mL/min. An average of 12 scans was performed at a repetition rate of 30 ms and a grid pulse width of 150 μs to build each spectrum. HS-GC-IMS data were obtained by Standalone (GAS, Dortmund, Germany), and the raw data were analyzed using VOCal (GAS, Dortmund, Germany) software to reveal information regarding the composition of the samples.

#### 2.3.3. Identification of Compounds

Compounds were identified by comparing their RIs and drift times with those of pure standards under the same conditions. To obtain more accurate results, all standard products were injected in batches. The information of the standards is shown in Table S2. The RIs were calculated using a mixture of C_4_–C_9_ ketones.

### 2.4. Statistical Analysis

To validate the models for Baijiu age identification, all samples were randomly divided into two categories: a test set and a prediction set. According the research of Gerhardt [30], a total of 35 samples were used as the test set and 4 samples were used as the prediction set. PLSR was used to establish a regression model between Baijiu ages (Y variable) and the volatiles (X variables), using the test set with SIMCA software (version 14.1 Umetrics; Sartorius Stedim Biotech AS; Umea, Sweden). The prediction ability of the model was validated using the prediction set. In this analysis, the data were subjected to Pareto scaling, wherein each variable was divided by the square root of its standard deviation.

To reduce the risk of overfitting, the number of latent variables in the model was decided by internal seven-fold cross-validation [31]. The samples were divided into seven groups to verify the accuracy of the model. The quality of the PLSR model was evaluated according to its R^2^Y and Q^2^ values, where R^2^Y represents the percentage of variation in the Y variable and Q^2^ represents the predictive ability [17]. Both values range from zero to one. Values closer to one indicate better goodness of fit and prediction ability. For the parameter Q^2^, values greater than 0.4 are acceptable. In addition, a permutation test was conducted to validate the robustness of the model [5].

## 3. Results and Discussion

### 3.1. Optimization of Headspace Parameters

Headspace conditions influence the response of HS-GC-IMS; therefore, researchers typically conduct optimization experiments before using HS-GC-IMS to analyze food samples, as has been conducted with olive oil, ham, and honey [17,18,19,26,32]. However, the optimization of distilled spirits by HS-GC-IMS has not been reported thus far. In this study, the headspace method parameters, including diluent, alcohol content, incubation temperature, and injection volume, were optimized to obtain more information, resolution, and signal intensity for the samples. To assess the effects of different parameters, Arroyo-Manzanares et al. relied on visual observation of topographic maps [17], and del Mar Contreras et al. used signal intensity [18]. In this study, three innovative indicators were used: the number, the height, and the volume of the peaks. The number of peaks represents the quantity of the detected compounds. The height and volume of the peaks reflect the concentration of the detected compounds. Below a certain concentration, the height and volume of the signals correspond to the concentration of the compound. However, when the concentration is greater than that value, the height of the signals remains unchanged and only the volume of the peaks increases. The number of protons provided by the tritium source was fixed; therefore, to obtain a better and more stable response from the low-concentration compounds during detection, the height and volume of the peaks were used as the second and third indicators, respectively.

#### 3.1.1. Effects of Salt Addition

**Figure 1:**
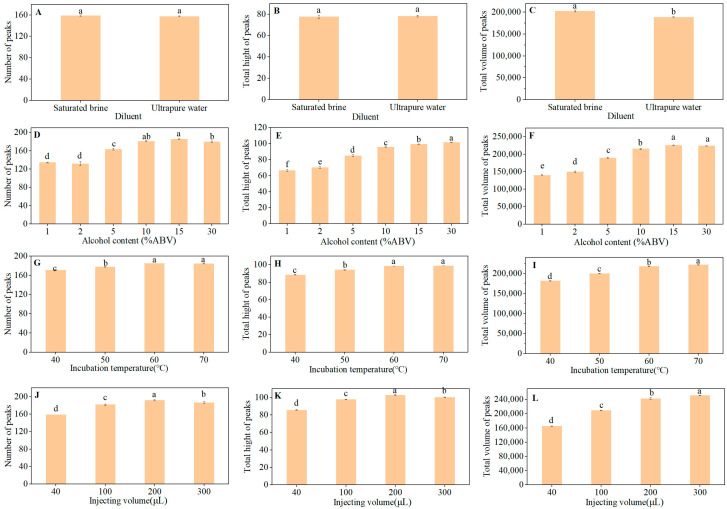
Number, total height, and total volume of signal peaks affected by diluent (**A**–**C**), alcohol content (**D**–**F**), incubation temperature (**G**–**I**), and injection volume (**J**–**L**). (Fisher’s LSD, significance level of 0.05.)

There are two opposite effects caused by the addition of NaCl, called salting in and salting out [18]. The addition of NaCl did not cause significant increases in the number and total height of the signals, but it did significantly increase the total volume of the peaks compared to that of the sample diluted with ultrapure water (Figure 1A–C and Figure S1). Owing to the salting out effect, the evaporation of volatile compounds from the solution to the headspace was promoted by the addition of salt [29].

#### 3.1.2. Effects of Alcohol Content

Ethanol content has two main effects on the detection of compounds. First, ethanol influences the release of other compounds [33]. Second, ethanol molecules compete with the compound molecules to have a fixed number of protons [20]. Thus, it was necessary to determine the appropriate alcohol content. Alcohol content had a significant impact on the HS-GC-IMS method (Figure 1D–F and Figure S2). The number of peaks was largest at 15% ethanol by volume (ABV), which is similar to the result at 10% ABV. However, the total height of the signals with 15% ABV was significantly higher than that of 10% ABV. Thus, each sample was diluted to 15% ABV for analysis, which is different from studies that used 10% ABV brandy with GC-IMS [5] and 5% ABV Baijiu with HS-SPME-GC-MS [34]. In order to ensure the consistency of the alcohol content, an accurate test of the alcohol content in each sample was carried out, and then each sample was diluted to 15% alcohol by volume strictly in accordance with the proportion.

#### 3.1.3. Effects of Incubation Temperature

As demonstrated in the present study, the incubation temperature directly affects the equilibrium concentration of volatile organic compounds in the headspace [35,36,37]. In this study, the incubation temperature was varied from 40 to 70 °C (Figure 1G–I and Figure S3). The incubation temperature was not raised above 70 °C to prevent water vapor from interfering with the operation of the instrument. The release of volatile organic compounds with particularly high boiling points was promoted by increasing the incubation temperature, which increased the intensity of the peaks [17]. For this reason, 70 °C was selected as the optimal condition.

#### 3.1.4. Effects of Injection Volume

The injection volume directly influences the concentration of volatile organic compounds entering the detector, making it an imperative parameter to optimize. The injection volume ranged from 40 to 300 μL. After considering all indicators, we determined that an injection volume of 200 μL was the most suitable (Figure 1J–L and Figure S4).

In summary, the final conditions were confirmed using a single-factor optimization experiment. The original Baijiu sample was diluted to 15% ABV with saturated brine, and each 20 mL vial was filled with 2 mL of the diluted sample. After incubation at 70 °C for 30 min, the autosampler sucked 200 μL of headspace gas into the chromatographic column for sample analysis.

### 3.2. Identification of Compounds in Baijiu Samples

**Figure 2:**
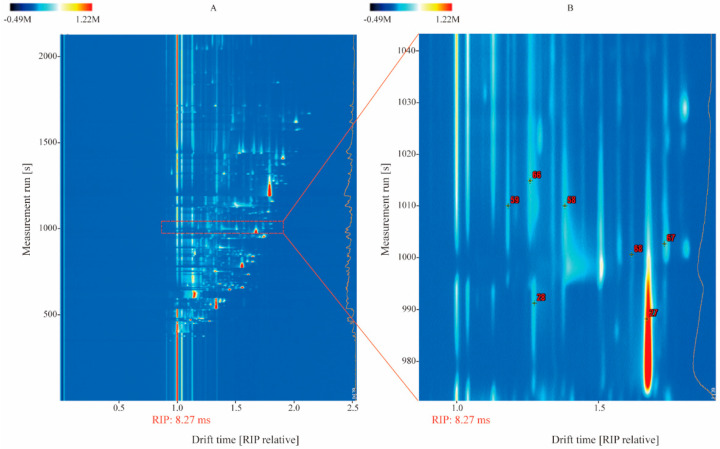
(**A**) Topographic plot of samples A1–4 detected by GC-IMS, and (**B**) detailed topographic plot of the signals in the red frame in (**A**).

Baijiu samples of different vintages were analyzed under the optimized conditions mentioned above. The results of the HS-GC-IMS analysis of samples A1–4 are shown as topographic plots where the x-axis represents the normalized drift time, and the y-axis represents the retention time (Figure 2A). The red vertical line denotes the normalized reaction ion peak. Each point in the plot represents one or multiple signals, and the different colors describe the intensity of the signals. Deeper red indicates a stronger intensity. Seventy-five percent of all signals appeared in the range of 1.0–2.0 ms with regard to normalized drift time and in the range of 500–1200 s with regard to retention time.

A total of 212 signals were detected in the Baijiu samples. A qualitative analysis led to the identification of the relationships between these signals and the ageing compounds in Baijiu. First, the RI of each compound was calculated using n-ketone. Then, the compounds were identified by comparing their RIs and drift times with those of the standard reference compounds, which are recorded in the NIST database and the IMS database. Thereafter, an IMS database of compounds in Baijiu was established (Table 1). A total of 93 compounds were identified in the samples, including 33 aldehydes and ketones, 39 esters, 18 alcohols, and 1 acid. Dimers and trimers were found in the high-concentration compounds. Notably, IMS provides the second separation of compounds, making it possible to separate isomers [20]. There were some separated isomers, e.g., code 22: 3-methyl-1-butanol and code 33: pentan-2-ol, with the formula of C_5_H_12_O.

Distinguishing samples using HS-GC-IMS has several advantages [20,38]. First, HS-GC-IMS is highly stable and sensitive and can accurately detect changes in compound concentrations. Second, HS-GC-IMS can achieve 2D separation of the signal peak, similar to two-dimensional gas chromatography and mass spectrometry (GC×GC-MS). Signal peaks that are originally clustered together can be distinguished to identify additional compounds by enlarging the gap between signals. This is useful to differentiate samples of various ageing durations. Because of the 2D separation ability of HS-GC-IMS, peaks 59 and 66 could be separated from each other and were identified as 1-butanol and 2-heptanone, respectively (Figure 2B).

**Figure 3:**
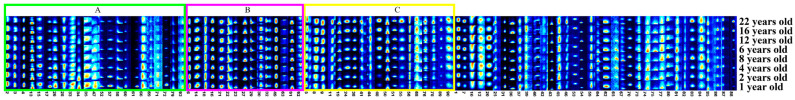
Gallery plot of the intensities of all the identified compounds in samples A1–A22 detected by GC-IMS. The codes of the compounds correspond to those in Table 1. (The intensities of the signal peaks in frame A generally decrease for each year; the intensities of the signal peaks in frame B have no obvious differences; the intensities of the signal peaks in frame C generally increase for each year).

A gallery plot was constructed of the voltage intensities of the 93 identified compounds (Figure 3). There are no obvious differences in the intensities of the signal peaks in frame B among samples of different age groups (Figure 3 frame B). In contrast, the intensities of the signal peaks in frame A generally decrease for each year (Figure 3 frame A). A trend of increasing signal intensity with age is apparent in frame C (Figure 3 frame C and Figure S5). The remaining peaks change irregularly with ageing time.

While some of the relationships between volatiles and ageing time were observable on a gallery plot, others required multivariate statistical analysis to differentiate and identify.

### 3.3. Establishment and Validation of Models for Baijiu Age Identification

The test set was used to establish a PLSR model for the identification of Baijiu age. Two data arrays were detected using HS-GC-IMS, including 212 signal peaks and 93 identified compounds. In some studies using GC-MS, UPLC-Orbitrap-MS/MS, GC-pulsed flame photometric detection, and GC-flame ionization detection, samples were discriminated [8,39,40]. With HS-GC-IMS, all of the signal peaks are usually used to establish the model because of the small number of identified compounds [18,24,26,41]. In this study, both signals and identified compounds were used to build models, and one data array was selected for in-depth analysis.

**Figure 4:**
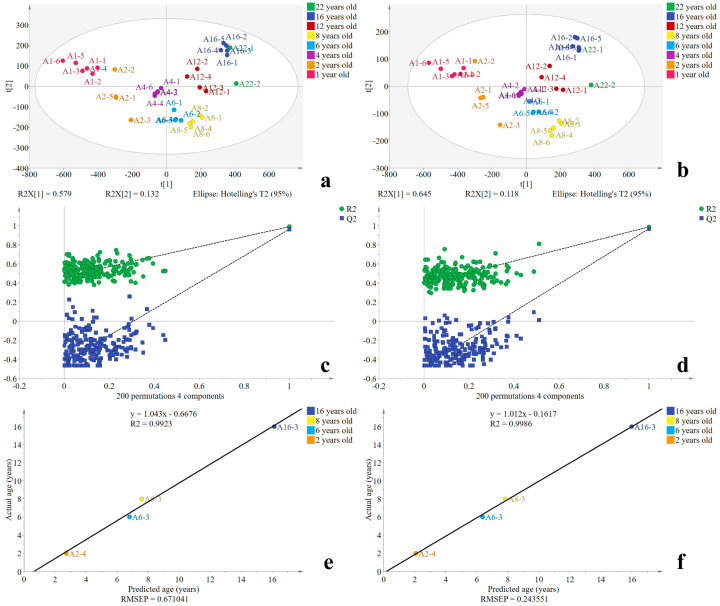
(**a**,**b**) Score scatter plots, (**c**,**d**) permutation test results (*n* = 200), and (**e**,**f**) graphs comparing the actual age and predicted age of the models. (**a**,**c**,**e**) Plots correspond to Model A. (**b**,**d**,**f**) Plots correspond to Model B.

The PLSR models based on 212 signal peaks and 93 identified compounds were named Model A and Model B, respectively. Four latent variables were selected to build the models in cases where the fifth latent variable was insignificant after seven-fold cross-validation. The value of Q^2^ was 0.962 in Model A and 0.968 in Model B. The value of R^2^Y was 0.990 in Model A and 0.988 in Model B. The values of both Q^2^ and R^2^Y were close to one, and there was little difference between the two models. This indicates that the optimized HS-GC-IMS conditions for untargeted analysis of samples can be used to distinguish samples from different years. The model has reliable predictive abilities and fit (Figure 4a,b), demonstrating that HS-GC-IMS has broad applications to sample differentiation.

A permutation test was performed to validate the robustness of the PLSR models (Figure 4c,d). This method involves running a random arrangement of sample data and then conducting statistical inference, which increases the number of samples fed into the model. This is particularly suitable for models with a small number of samples. The result was obtained through 200 permutation tests using SIMCA software. In Figure 4c,d, all the Q^2^ values (blue) and R^2^ values (green) to the left are lower than the original points to the right. Moreover, the regression line of the Q^2^ points intersects the y-axis below zero. Therefore, neither model has a risk of overfitting, which indicates that both models are valid.

The PLSR models for Baijiu age identification were established based on the sufficient Baijiu samples from different years having different concentrations of aroma compounds. A connection was built between the age of the Baijiu and the concentration of aroma compounds, which made it feasible to use the model to identify the age of Baijiu.

To more accurately understand the predictive ability of the established PLSR model, the prediction set (four Baijiu samples assumed to have unknown ages) was used to verify the model (Figure 4e,f). With a reliable model, all the points should fall close to the 45° line through the origin, and the prediction set should be close to the regression line. The goodness of fit value (R^2^) of the regression line indicates the fitness level. The closer the R^2^ to one, the better the fit of the model. The value of R^2^ was 0.9923 in Model A and 0.9986 in Model B. The root mean square error of prediction (RMSEP) of Model A was larger than that of Model B, being 0.671 and 0.244, respectively. In addition, as shown in Supplementary Table S3, the deviation of Model B was smaller than that of Model A, implying that Model B has a stronger predictive ability than that of Model A. HS-GC-IMS was sensitive to aldehydes, ketones, esters, and alcohols; thus, many of these substances were detected. In addition, previous studies have shown that alcohols, esters, aldehydes, and ketones undergo significant changes during the Baijiu ageing process [8,12,42]. Therefore, analyzing the changes in these compound concentrations can distinguish and identify the age of the samples.

In summary, PLSR Model B, based on 93 identified compounds, had better fitting and predictive abilities and more accurately distinguished Baijiu samples from different vintages and identified their ages. It is worth noting that the method can also be applied to other alcoholic beverages based on analyzing sufficient numbers of samples to distinguish and identify the age of unknown samples.

**Figure 5:**
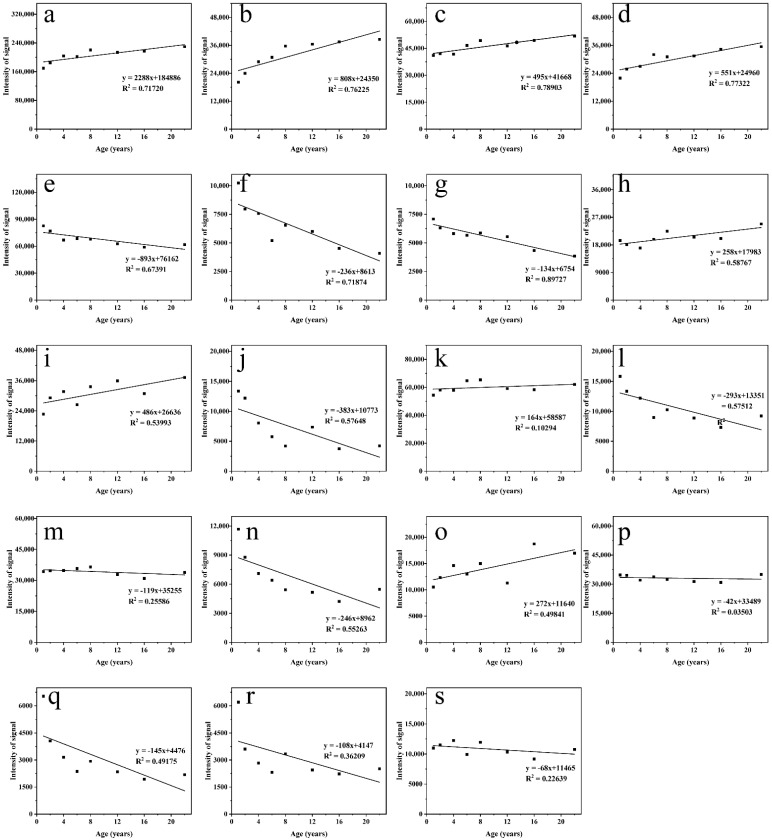
Scatter plots of the changes in voltage intensity of ethyl hexanoate ^A^ (**a**), propyl hexanoate ^A^ (**b**), ethyl pentanoate ^A^ (**c**), ethyl heptanoate ^A^ (**d**), ethyl acetate ^A^ (**e**), 2-methyl-1-propanol (**f**), methylpropanal ^B^ (**g**), butan-2-ol ^A^ (**h**), octanoic acid ethyl ester ^B^ (**i**), isoamyl acetate ^A^ (**j**), ethyl butyrate ^A^ (**k**), nonanal (**l**), ethyl hexanoate (**m**), ethyl lactate (**n**), 2-methyl butanoic acid ethyl ester ^A^ (**o**), 3-methyl-1-butanol (**p**), octanal (**q**), furfural ^A^ (**r**), and 1-hexanol ^A^ (**s**).

According to Model B, there were 19 compounds with variable importance for prediction (VIP) scores greater than one. These 19 compounds (Figure 5), including ethyl hexanoate ^A^, propyl hexanoate ^A^, ethyl pentanoate ^A^, ethyl heptanoate ^A^, ethyl acetate ^A^, 2-methyl-1-propanol, methylpropanal ^B^, butan-2-ol ^A^, octanoic acid ethyl ester ^B^, isoamyl acetate ^A^, ethyl butyrate ^A^, nonanal, ethyl hexanoate, ethyl lactate, 2-methyl butanoic acid ethyl ester ^A^, 3-methyl-1-butanol, octanal, furfural ^A^, and 1-hexanol ^A^, were most important for identifying the ages of samples. Fifty-eight percent of the total compounds in Baijiu were esters, which illustrates that these important flavor compounds play a crucial role in establishing a regression model for Baijiu age [8,12,41]. The remaining compounds with VIP scores greater than one were alcohols and aldehydes, accounting for a combined 21% of the 19 compounds. Overall, HS-GC-IMS exhibited outstanding performance at identifying the sample age, implying that fewer compounds can be used in future tests to make it more rapid. Therefore, it is reasonable to apply HS-GC-IMS to age Baijiu.

Ethyl hexanoate ^A^, propyl hexanoate ^A^, ethyl pentanoate ^A^, and ethyl heptanoate ^A^ (Figure 5a–d) were positively correlated with ageing time, while ethyl acetate ^A^, 2-methyl-1-propanol, and methylpropanal ^B^ (Figure 5e,f) were negatively correlated with ageing time. The R^2^ values for these compounds were greater than 0.65. The remaining compounds (Figure 5g–s) play an important role in the discrimination of the ageing year, but have no linear correlation with Baijiu age, exhibiting R^2^ values of less than 0.6.

The change in compounds is also affected by the ageing method, manufacturer, and storing conditions, which may reduce the accuracy of the prediction. In future study, a larger number of samples will be collected to improve the accuracy of the prediction. In the study, the voltage intensity of the compound was used to identify the age of the Baijiu. However, it is important to determine the absolute concentration of compounds so that the age of samples from different batches can be identified. Thus, the determination of absolute concentration is part of our next plan.

## 4. Conclusions

This study demonstrated the potential of HS-GC-IMS to discriminate Baijiu of different ages. HS-GC-IMS combined with PLSR performed excellently in distinguishing Baijiu samples of different ages. PLSR Model A, based on 212 signal peaks, and PLSR Model B, based on 93 identified compounds, were both valid; however, Model B more accurately identified the ages of unknown Baijiu samples, exhibiting R^2^ value of 0.9986 and RMSEP of 0.244. HS-GC-IMS combined with PLSR also has better accuracy and precision for age detection than other methods. There were 19 compounds with VIP scores greater than one in Model B, including 11 esters, 4 alcohols, 4 aldehydes, and 1 acid. Among them, seven compounds are potential ageing markers in Baijiu samples, which are positively or negatively correlated with ageing time. Consequently, HS-GC-IMS combined with PLSR can be used to rapidly and accurately identify the age of Baijiu.

## Figures and Tables

**Table 1:** Information about the identified volatiles in Baijiu samples detected by GC-IMS.

Number	Codes	Compound	RI ^(4)^	Rt (s) ^(5)^	Dt (RIP Relative) ^(6)^	Location in Figure 3
**Aldehydes and Ketones**						
1	21	(E)-2-hexenal	1210	1124	1.183	frame B
2	20	(E)-2-hexenal ^A (1)^	1210	1124	1.512	
3	46	(E,Z)-2,6-nonadienal	1590	2051	1.384	
4	65	1,1-diethoxyethane	902	545	1.069	
5	64	1,1-diethoxyethane ^A^	902	545	1.204	
6	66	2-heptanone	1163	1015	1.261	frame A
7	68	2-heptanone ^A^	1157	1001	1.618	frame C
8	67	2-heptanone ^B (2)^	1158	1003	1.734	
9	9	2-methylbutanal	933	584	1.404	frame C
10	14	2-pentanone	931	582	1.375	frame B
11	11	3-methylbutanal	923	571	1.164	frame C
12	10	3-methylbutanal ^A^	923	571	1.396	
13	87	benzaldehyde	1518	1838	1.147	
14	86	benzaldehyde ^A^	1518	1839	1.267	
15	88	benzaldehyde ^B^	1518	1838	1.461	
16	89	benzaldehyde ^C (3)^	1518	1838	1.649	frame C
17	90	butan-2-one	915	561	1.059	frame C
18	48	butan-2-one ^A^	914	561	1.242	frame C
19	63	butyraldehyde	889	530	1.097	frame B
20	8	butyraldehyde ^A^	889	530	1.132	frame C
21	7	butyraldehyde ^B^	889	530	1.275	
22	60	furfural	1473	1716	1.08	frame A
23	61	furfural ^A^	1472	1715	1.325	frame A
24	42	hexanal	1100	875	1.262	
25	41	hexanal ^A^	1100	875	1.556	frame C
26	6	methylpropanal	830	464	1.112	frame B
27	5	methylpropanal ^A^	830	464	1.117	frame C
28	4	methylpropanal ^B^	830	464	1.277	frame A
29	79	nonanal	1361	1449	1.565	frame A
30	78	nonanal ^A^	1361	1449	1.961	frame C
31	72	octanal	1302	1324	1.401	frame A
32	47	propan-2-one	849	471	1.117	frame A
33	3	propionaldehyde	816	450	1.073	frame A
34	1	propionaldehyde ^A^	816	450	1.142	
35	2	propionaldehyde ^B^	816	450	1.166	frame A
**Esters**						
36	62	1-methylethyl acetate	841	476	1.165	
37	37	2-methyl butanoic acid ethyl ester	1084	841	1.259	
38	36	2-methyl butanoic acid ethyl ester ^A^	1084	841	1.647	
39	71	3-methylbutyl butanoate	1275	1265	1.405	frame A
40	70	3-methylbutyl butanoate ^A^	1274	1261	1.932	
41	54	3-methylbutyl hexanoate	1463	1672	2.127	
42	40	butyl acetate	1087	846	1.62	
43	39	ethyl 3-methylbutanoate	1070	811	1.246	
44	38	ethyl 3-methylbutanoate ^A^	1070	811	1.646	frame C
45	23	ethyl 4-methylpentanoate	1198	1098	1.774	frame B
46	12	ethyl acetate	909	554	1.097	
47	13	ethyl acetate ^A^	909	554	1.333	frame A
48	92	ethyl butyrate	1065	800	1.204	frame B
49	91	ethyl butyrate ^A^	1065	800	1.557	frame B
50	18	ethyl heptanoate	1350	1426	1.41	frame B
51	19	ethyl heptanoate ^A^	1350	1426	1.907	frame C
52	56	ethyl hexanoate	1249	1210	1.339	frame C
53	50	ethyl hexanoate ^A^	1253	1220	1.782	frame C
54	15	ethyl isobutyrate	990	664	1.312	frame A
55	16	ethyl isobutyrate ^A^	991	664	1.312	frame B
56	17	ethyl lactate	1357	1441	1.531	frame A
57	28	ethyl pentanoate	1153	991	1.276	frame A
58	27	ethyl pentanoate ^A^	1153	991	1.672	frame B
59	77	propyl hexanoate	1327	1377	1.398	
60	76	propyl hexanoate ^A^	1327	1375	1.855	frame C
61	51	propyl hexanoate ^B^	1327	1380	1.91	frame C
62	35	isoamyl acetate	1133	945	1.304	frame A
63	34	isoamyl acetate ^A^	1133	945	1.745	frame A
64	31	isobutyl acetate	1031	734	1.604	
65	53	methyl 2-methylbutanoate	1022	723	1.533	
66	25	methyl hexanoate	1195	1093	1.285	
67	24	methyl hexanoate ^A^	1195	1093	1.67	frame C
68	45	octanoic acid ethyl ester	1438	1627	1.476	
69	44	octanoic acid ethyl ester ^A^	1437	1626	1.905	frame C
70	43	octanoic acid ethyl ester ^B^	1437	1626	2.017	
71	69	pentyl acetate	1183	1063	1.312	frame A
72	57	pentyl acetate ^A^	1181	1061	1.754	frame A
73	55	pentyl butanoate	1328	1382	1.955	frame C
74	49	propanoic acid ethyl ester	978	648	1.45	frame B
**Alcohols**						
75	74	1-hexanol	1361	1450	1.321	
76	73	1-hexanol ^A^	1360	1447	1.657	
77	75	1-hexanol ^B^	1360	1447	1.761	
78	85	1-octanol	1523	1853	1.301	
79	84	1-octanol ^A^	1523	1852	1.83	
80	83	1-octanol ^B^	1522	1850	1.869	
81	26	2-methyl-1-propanol	1089	851	1.15	frame A
82	22	3-methyl-1-butanol	1216	1136	1.503	frame B
83	59	1-butanol	1161	1010	1.184	
84	58	1-butanol ^A^	1161	1010	1.383	frame A
85	30	butan-2-ol	1042	755	1.23	frame B
86	29	butan-2-ol ^A^	1042	755	1.33	frame B
87	81	heptan-1-ol	1417	1577	1.398	
88	80	heptan-1-ol ^A^	1417	1578	1.472	
89	82	linalool	1543	1908	1.253	
90	33	pentan-2-ol	1132	944	1.287	frame A
91	32	pentan-2-ol ^A^	1132	944	1.451	frame B
92	93	terpinen-4-ol	1589	2048	1.221	frame A
**Acid**						
93	52	acetic acid	1467	1680	1.152	frame A

(1) Superscript A means that the compound is a dimer; (2) superscript B means that the compound is a trimer; (3) superscript C means that the compound is a tetramer; (4) RI, retention index of the compound; (5) Rt, retention time of the compound; (6) Dt, drift time of the compound.

## Data Availability

The data that support the findings of this study are available from the corresponding author upon request.

## Supplementary Materials

The following are available online at https://www.mdpi.com/article/10.3390/foods10112888/s1, Figure S1: Topographic plot of Baijiu samples diluted with saturated brine (A), ultrapure water (B) detected by GC-IMS, Figure S2: Topographic plot of Baijiu samples diluted in different alcohol content detected by GC-IMS, Figure S3: Topographic plot of Baijiu samples detected by GC-IMS in different incubation temperature, Figure S4; Topographic plot of Baijiu samples detected by GC-IMS with different injection volume, Figure S5: Topographic plot of Baijiu samples with different years detected by GC-IMS, Table S1: Baijiu samples, Table S2: The information of standards, Table S3: The age discrimination of the prediction set samples.
