# Supplementary material for: Untargeted Headspace-Gas Chromatography-Ion Mobility Spectrometry in Combination with Chemometrics for Detecting the Age of Chinese Liquor (Baijiu)
Source: Foods. 2021 Nov 22;10(11):2888. doi: 10.3390/foods10112888 (PMC8621296; doi:10.3390/foods10112888)
Supplement: Supplementary file 1 [file foods-10-02888-s001.zip › foods-1454538-supplementary.pdf]

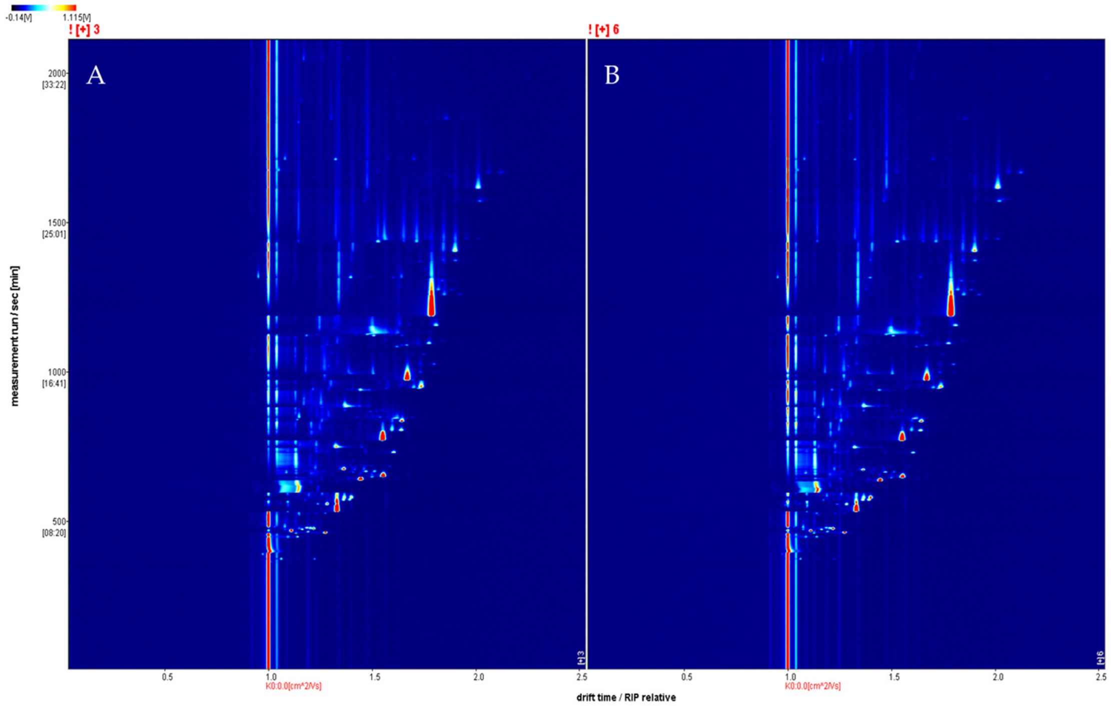

Figure S1. Topographic plot of Baijiu samples diluted with saturated brine (A), ultrapure water (B) detected by GC-IMS.

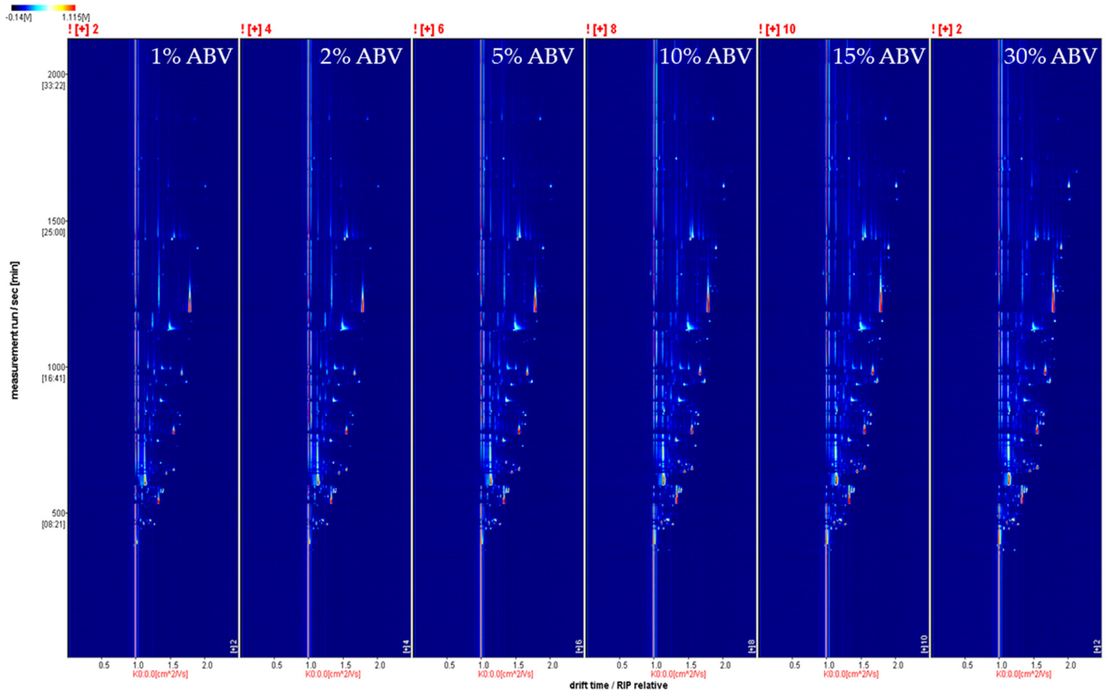

Figure S2. Topographic plot of Baijiu samples diluted in different alcohol content detected by GC-IMS.

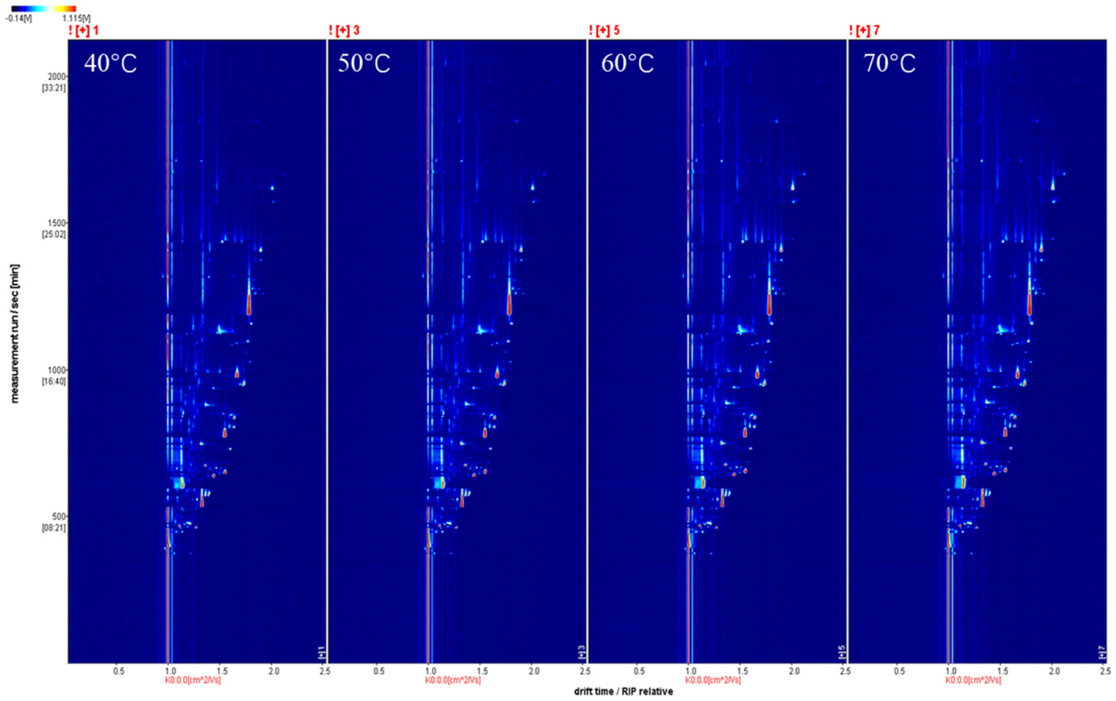

Figure S3. Topographic plot of Baijiu samples detected by GC-IMS in different incubation temperature.

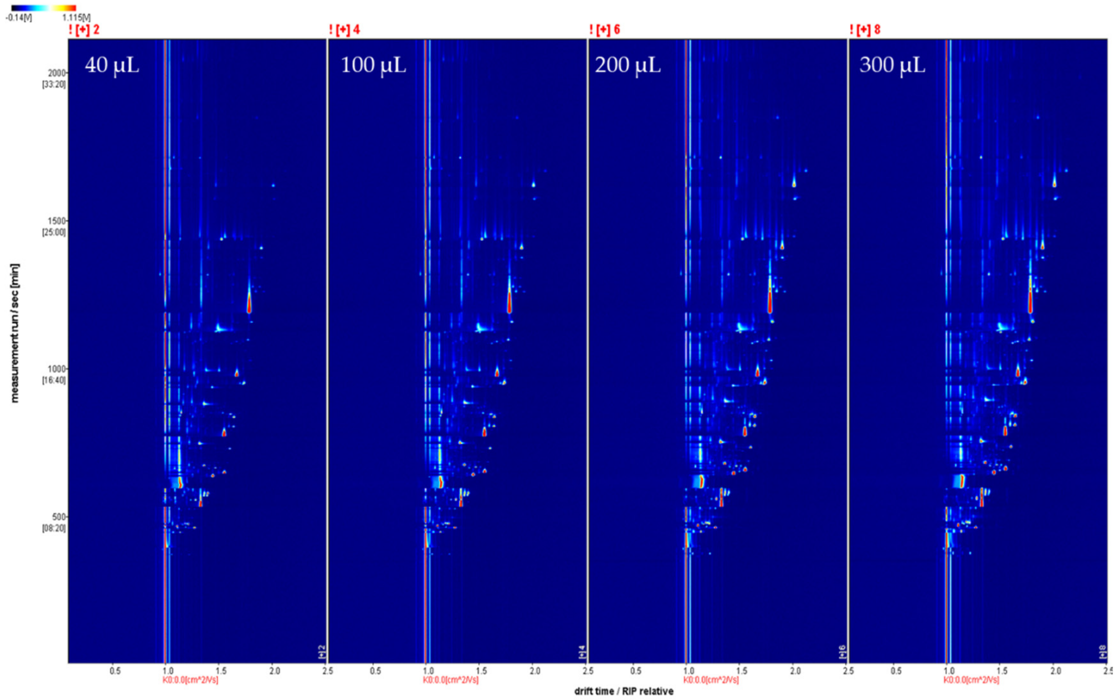

Figure S4. Topographic plot of Baijiu samples detected by GC-IMS with different injection volume.

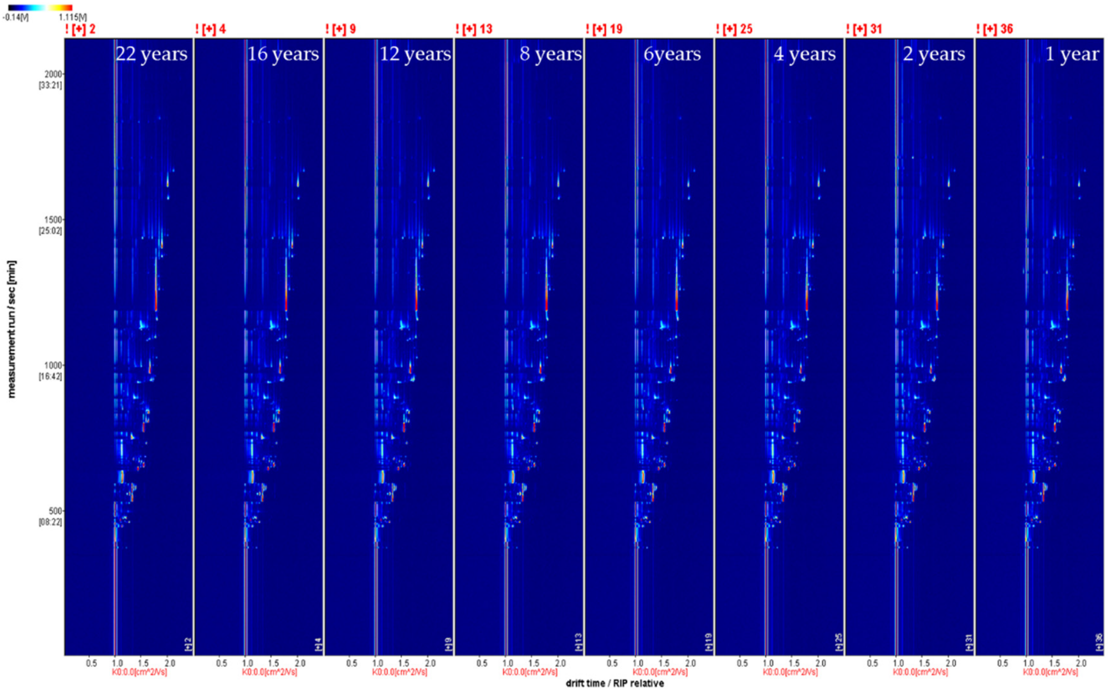

Figure S5. Topographic plot of Baijiu samples with different years detected by GC-IMS.

Table S1 Baijiu samples

Codes	age (years)	Vintages	Alcohol content (%)
A22-1	22	1998	61.5
A22-2	22	1998	68.8
A16-1	16	2004	61.2
A16-2	16	2004	60.9
A16-3	16	2004	61.1
A16-4	16	2004	61
A16-5	16	2004	60.5
A12-1	12	2008	65.7
A12-2	12	2008	61.6
A12-3	12	2008	64
A12-4	12	2008	63.2
A8-1	8	2012	67.9
A8-2	8	2012	67.5
A8-3	8	2012	67.6
A8-4	8	2012	68.7

A8-5	8	2012	68.5
A8-6	8	2012	68.8
A6-1	6	2014	65.7
A6-2	6	2014	67
A6-3	6	2014	67.1
A6-4	6	2014	67.1
A6-5	6	2014	66.8
A4-1	4	2016	64.6
A4-2	4	2016	64.7
A4-3	4	2016	65.1
A4-4	4	2016	65.2
A4-5	4	2016	65.3
A4-6	4	2016	65
A2-1	2	2018	68.5
A2-2	2	2018	62.1
A2-3	2	2018	69.2
A2-4	2	2018	68.5
A2-5	2	2018	66.9
A1-1	1	2019	67.7
A1-2	1	2019	68.2
A1-3	1	2019	68.8
A1-4	1	2019	68.9
A1-5	1	2019	69
A1-6	1	2019	69.2

Table S2 The information of standards

Number	Compound	Batch
1	1,1-diethoxyethane	1
2	hexanal	1
3	1-methylethyl acetate	1
4	ethyl hexanoate	1
5	ethyl lactate	1
6	1-octanol	1

7	heptan-1-ol	1
8	2-methylbutanal	2
9	3-methylbutanal	2
10	butan-2-one	2
11	propan-2-one	2
12	propionaldehyde	2
13	3-methylbutyl hexanoate	2
14	ethyl 3-methylbutanoate	2
15	ethyl 4-methylpentanoate	2
16	propyl hexanoate	2
17	2-methyl-1-propanol	2
18	butan-2-ol	2
19	terpinen-4-ol	2
20	benzaldehyde	3
21	octanal	3
22	2-methyl butanoic acid ethyl ester	3
23	ethyl heptanoate	3
24	methyl 2-methylbutanoate	3
25	pentyl acetate	3
26	1-hexanol	3
27	1-butanol	3
28	linalool	3
29	acetic acid	3
30	2-pentanone	4
31	methylpropanal	4
32	nonanal	4
33	3-methylbutyl butanoate	4
34	ethyl isobutyrate	4
35	isoamyl acetate	4
36	isobutyl acetate	4
37	methyl hexanoate	4
38	3-methyl-1-butanol	4
39	pentan-2-ol	4
40	(E)-2-hexenal	5

41	(E, Z)-2,6-nonadienal	5
42	2-heptanone	5
43	butyraldehyde	5
44	furfural	5
45	butyl acetate	5
46	ethyl acetate	5
47	ethyl butyrate	5
48	ethyl pentanoate	5
49	octanoic acid ethyl ester	5
50	pentyl butanoate	5
51	propanoic acid ethyl ester	5

Table S3 The age discrimination of the prediction set samples

Sample	Ture/year	Model A		Model B	
		Predicted	Deviation	Predicted	Deviation
A2-4	2	1.4184	-0.5816	1.8623	-0.1377
A6-3	6	5.5904	-0.4096	5.9103	-0.0897
A8-3	8	7.6764	-0.3236	7.9343	-0.0657
A16-3	16	16.0204	0.0204	16.0303	0.0303
